# BNC2 as a putative transcriptional coordinator linking energy state to neural circuits

**DOI:** 10.1016/j.isci.2026.115733

**Published:** 2026-04-17

**Authors:** Zixu Zhang, Shengru Hu, Kaiyi Hua, Peng Li, Yike Duan, Wangqi Shao, Shihao Sun, Hongbo Zhang, Mingdao Mu

**Affiliations:** 1 Beijing Life Science Academy (BLSA), Beijing 102209, China; 2 School of Medicine, Southeast University, 87 Dingjiaqiao Road, Nanjing, P.R. China; 3 The Key Laboratory of Developmental Genes and Human Disease, Ministry of Education, The School of Life Science and Technology, Southeast University, 2 Sipailou Road, Nanjing, P.R. China; 4 Key Laboratory of Synthetic Biology of Ministry of Agriculture and Rural Affairs, Tobacco Research Institute, Chinese Academy of Agricultural Sciences, Qingdao 266101, China

**Keywords:** Molecular biology, Neuroscience, Systems biology

## Abstract

Energy homeostasis relies on interconnected brain circuits that integrate hormonal signals with behavioral outputs. Building upon the established roles of canonical transcriptional regulators like STAT3 and FOXO1 in metabolic neurons, we synthesize emerging evidence positioning basonuclin-2 (BNC2), a deeply conserved zinc-finger transcription factor, as a putative coordinator of energy balance. Spatial transcriptomic mapping highlights selective BNC2 expression within the hypothalamic arcuate nucleus and ventral pallidum. Recent functional studies demonstrate that hypothalamic BNC2 neurons suppress appetite via leptin-driven GABAergic inhibition of AgRP/NPY neurons, whereas ventral pallidum BNC2 neurons modulate reward-driven food intake. Together, these findings suggest BNC2 functions as a critical bridge between homeostatic and hedonic feeding circuits. Future investigations addressing isoform specificity, direct hormonal sensing, and human genetic relevance will be essential to evaluate BNC2 as a therapeutic target for obesity and related metabolic disorders.

## Graphical abstract

**Figure:**
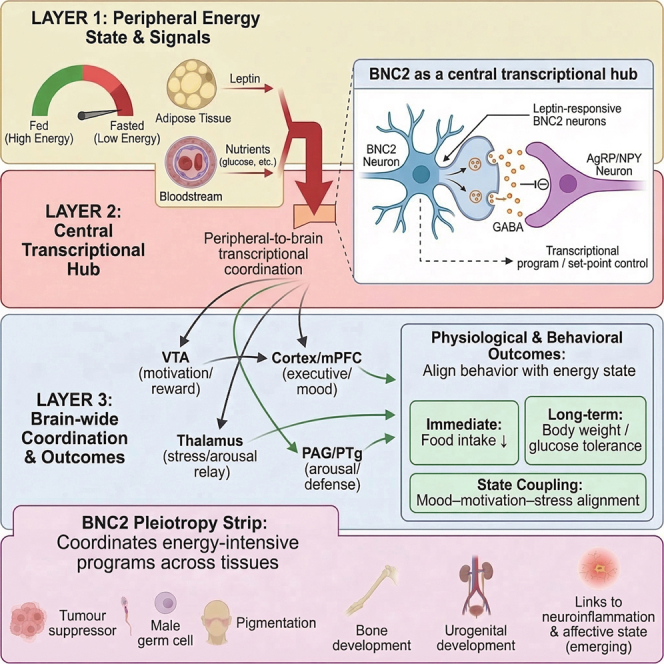

## Introduction

### The context: Transcriptional control of metabolic neural circuits

The escalating global prevalence of obesity and its associated metabolic disorders, such as type 2 diabetes, represent one of the preeminent public health challenges of our time.^1^ At the heart of this crisis lies a dysregulation of energy homeostasis, a complex physiological process exquisitely orchestrated by the brain.^2^ For decades, research has illuminated the roles of key neuropeptide systems (e.g., AgRP and POMC) and hormonal pathways (e.g., leptin and insulin) in controlling energy balance.^2^^,^^3^ Crucially, seminal work has identified master transcriptional regulators, such as STAT3, FOXO1, CREB, and BSX, that function as the downstream effectors of these signaling cascades.^4^ These giants of metabolic regulation provide the foundational machinery for cellular response.

However, the intricate adaptability of the metabolic system, which must coordinate acute appetite suppression with long-term structural plasticity and behavioral state, suggests the existence of additional, perhaps distinctively localized, regulatory layers. Beyond these canonical gatekeepers, identifying auxiliary transcriptional layers is essential to fully decode the neurobiological plasticity required for long-term metabolic adaptation.^3^ In this context, basonuclin-2 (BNC2) has emerged as a putative candidate for such a foundational metabolic transcriptional component.^5^

BNC2 is one of the most evolutionarily conserved members of the C2H2 zinc finger protein family.^6^ Its extraordinary sequence conservation (97.2% identity between human and mouse) strongly suggests it performs fundamental biological functions under intense selective pressure.^6^ This profound conservation stands in fascinating contrast to its structural complexity. The human *BNC2* gene possesses 6 promoters and 23 alternatively spliced exons, theoretically capable of generating nearly 90,000 mRNA isoforms.^7^ While this vast regulatory potential poses challenges for functional dissection (as discussed in Section 6), it also suggests BNC2 may coordinate complex, multi-system physiological programs, making it an ideal candidate for orchestrating the intricate metabolic control networks of the brain. Unlike BNC1, BNC2 maintains consistent nuclear localization and co-localizes with splicing factors, suggesting functions that may extend beyond canonical transcriptional regulation.^8^ This duality positions BNC2 as a potentially pivotal regulator in complex physiological processes, including metabolism.

### Precedents from the periphery: BNC2 in adaptive responses and tissue maintenance

Before its neural metabolic functions were discovered, BNC2 established itself as a regulator of essential adaptive and maintenance processes in peripheral tissues, providing important precedents for its role in central energy homeostasis.

Pigmentation and environmental adaptation: BNC2 has emerged as a key genetic determinant of human skin pigmentation traits, a process linked to vitamin D synthesis, which itself plays a role in metabolic health. Genome-wide association studies (GWASs) have consistently identified *BNC2* variants associated with skin color variation in European populations,^9^ facial pigmented spots in Korean and Japanese women,^10^^,^^11^ and freckle occurrence in Spanish populations.^12^ This process of pigment production and cellular maintenance represents a critical adaptive response to environmental stress (e.g., UV radiation) requiring precise transcriptional tuning. Notably, BNC2 has been implicated in skin cancer susceptibility, with protective alleles associated with reduced risk of cutaneous squamous cell carcinoma^13^ and actinic keratosis.^14^ In zebrafish, *bnc2* plays a crucial non-autonomous role in promoting pigment cell survival.^15^

Developmental processes and tissue homeostasis: the requirement for BNC2 is particularly evident in developmental programs characterized by high cellular turnover and strict homeostatic requirements, such as spermatogenesis, urogenital development, and skeletal growth.^16^^,^^17^^,^^18^^,^^19^ In male germ cell development, BNC2 is essential for preventing premature meiotic initiation, highlighting its role in maintaining cellular state decisions.^16^ The protein is also crucial for urogenital development^17^^,^^18^ and skeletal development.^19^

Cancer biology and the obesity link: BNC2 has been extensively studied as a tumor suppressor across multiple cancer types.^20^^,^^21^^,^^22^^,^^23^ This role is particularly relevant to the perspective of metabolic regulation. Given that metabolic reprogramming (the Warburg effect) is a hallmark of cancer, BNC2’s involvement in tumor suppression suggests it may govern the resource allocation required for cell survival.^24^^,^^25^ Furthermore, this dual role raises an intriguing possibility: BNC2 could function as a molecular link in obesity-associated carcinogenesis.

This established precedent of BNC2 as an integrator coordinating tissue adaptation and maintenance begs a compelling question: does this coordinating role extend into the CNS to directly regulate systemic metabolism?

## Mapping the BNC2 expression landscape: An anatomical roadmap for hypothesis generation

**Figure 1:**
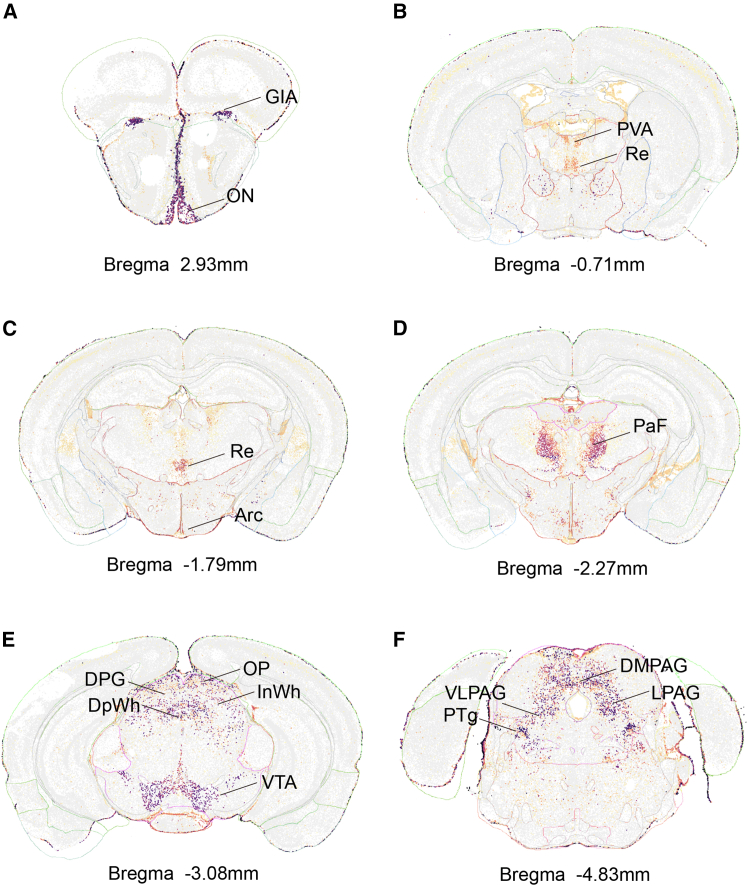
Spatial transcriptomic mapping of Bnc2 mRNA distribution in the mouse brain Representative coronal sections derived from the high-resolution MERFISH dataset (Allen Brain Cell Atlas) illustrating the region-specific expression patterns of *Bnc2*. (A) Anterior section (Bregma +2.93 mm) showing expression restricted to the glomerular layer of the accessory olfactory bulb (GlA) and olfactory nerve layer (ON). (B) Thalamic section (Bregma -0.71 mm) exhibiting expression in the anterior paraventricular thalamic nucleus (PVA) and reuniens nucleus (Re). (C) Mid-thalamic and hypothalamic level (Bregma -1.79 mm) highlighting expression in the Re and the arcuate hypothalamic nucleus (Arc). (D) Posterior thalamus (Bregma -2.27 mm) displaying notably dense expression in the parafascicular nucleus (PaF). (E) Midbrain section (Bregma -3.08 mm) showing expression in the ventral tegmental area (VTA) and distinct layers of the superior colliculus (deep gray [DpG], deep white [DpWh], optic nerve [Op], and intermediate white [InWh] layers). (F) Brainstem section (Bregma -4.83 mm) demonstrating distributed expression across the periaqueductal gray (dorsomedial [DMPAG], ventrolateral [VLPAG], and lateral [LPAG] subdivisions) and the pedunculotegmental nucleus (PTg). Data source: Allen Brain Cell Atlas (knowledge.brain-map.org).

The leap from understanding BNC2’s peripheral functions to uncovering its central nervous system roles has been sparked by advances in spatial transcriptomics. For BNC2 specifically, the application of high-resolution multiplexed error-robust fluorescence *in situ* hybridization (MERFISH) to whole-brain analysis has provided the first comprehensive map of its neural expression patterns.^26^^,^^27^ This technological leap revealed that BNC2 is not broadly expressed throughout the brain, but instead shows a highly heterogeneous and region-specific distribution, with expression appearing clustered in specific neural nuclei (Figure 1).

**Figure 2:**
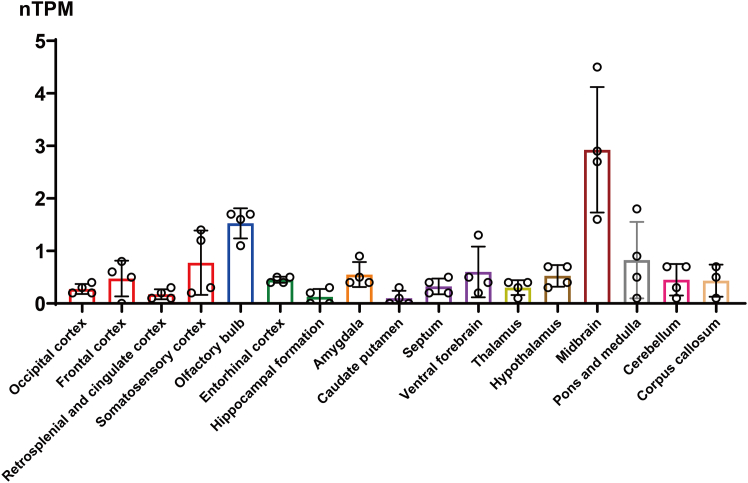
Regional heterogeneity of Bnc2 mRNA abundance in the mouse brain Quantitative analysis of *Bnc2* expression levels (normalized transcripts per million, nTPM) across distinct brain regions, sourced from The protein atlas bulk RNA-seq dataset.^28^ Anatomical structures are color-coded and grouped into major divisions: cerebral cortex, olfactory bulb, hippocampal formation, amygdala, basal ganglia, thalamus, hypothalamus, midbrain, pons and medulla, cerebellum, and corpus callosum (white matter). Bars represent the mean nTPM value, and dots indicate individual sample replicates, demonstrating expression variability within each region. Data are presented as mean ± SD.

However, interpreting this expression atlas requires acknowledging several critical caveats, which are central to framing this work as a perspective rather than a definitive review. First, the assertion of enrichment in any given nucleus is, at present, a qualitative observation based on visual inspection of cell clustering. Rigorous statistical analysis, normalized to local cell density, is needed to confirm these patterns. Second, the reliance on a single MERFISH dataset means these findings await validation from independent spatial transcriptomic platforms (e.g., slide-seq, Visium) or traditional *in situ* hybridization to rule out platform-specific artifacts. To address this, we cross-referenced MERFISH data with independent bulk RNA-seq data from The human protein atlas (Figure 2). The general regional distribution observed in MERFISH is consistent with the bulk RNA-seq data, which also shows elevated expression in the hypothalamus, basal ganglia, and thalamus, supporting the biological validity of the overall anatomical pattern. Third, MERFISH probes are typically designed against common exons. Given the vast splicing potential of *BNC2*, the current spatial maps likely reflect total *Bnc2* mRNA abundance rather than distinguishing specific functional splice variants.

Despite these limitations, the observed expression pattern provides a compelling anatomical foundation for generating new hypotheses. The MERFISH data show BNC2 expression in key nodes of metabolic control, including the arcuate nucleus of the hypothalamus (Arc), and in circuits related to reward and motivation, such as the ventral tegmental area (VTA). Furthermore, the expression extends to sensory and integration hubs like the thalamus (e.g., PVA, Re, and PaF) and superior colliculus (e.g., DpG and Op) (Figure 1).

This sparse-but-distributed expression signature suggests BNC2 is positioned to act as a multi-system coordinator. To translate this anatomical map into a functional framework, we summarize the potential implications in Table 1. Here, we link the brain regions with BNC2 expression to their known functions, thereby proposing “hypothesized functions” for BNC2 in metabolism-focused behaviors. For instance, its presence in the hypothalamus has an established role in homeostatic feeding, while its expression in limbic and brainstem regions suggests hypothesized functions in reward-seeking and arousal.

**Table 1:** Regional distribution of BNC2 expression and functional implications

Brain region	Specific expression sites	Known regional functions	Hypothesized roles of BNC2-Expressing Neurons (requires validation)	Reference
Olfactory System	glomerular layer (accessory olfactory bulb), olfactory nerve layer	pheromone processing, social behavior, olfactory signal transmission	hypothesis: modulating olfactory-guided feeding behaviors based on metabolic state	^29^^,^^30^^,^^31^
Thalamus	paraventricular nucleus (anterior), reuniens nucleus, parafascicular nucleus	stress responses, hippocampal-prefrontal relay, arousal regulation	hypothesis: integrating visceral metabolic signals with cortical decision-making on feeding	^32^^,^^33^^,^^34^^,^^35^^,^^36^^,^^37^
Hypothalamus	arcuate nucleus	energy homeostasis, leptin signaling, appetite control	established role: direct, leptin-mediated appetite suppression and long-term metabolic regulation	^5^
Superior Colliculus	deep gray layer, optic nerve layer, deep/intermediate white layers	visual-motor integration, attention, orienting behaviors	hypothesis: guiding foraging-related orienting behaviors and visual attention to food cues	^38^^,^^39^^,^^40^
Limbic System	ventral Pallidum	reward valuation, hedonic feeding	established role: modulating high-fat diet intake and body weight gain	^41^^,^^42^
Brainstem	ventral tegmental area	reward processing, motivation, dopamine signaling	hypothesis: modulating food reward valuation and motivation for food-seeking	^43^^,^^44^^,^^45^
	periaqueductal gray, pedunculotegmental nucleus	defensive behaviors, pain modulation, sleep-wake control	hypothesis: coordinating arousal state with metabolic needs, e.g., promoting wakefulness during fasting	^46^^,^^47^^,^^48^

## Functional dissection of BNC2-expressing neural circuits

### A mechanistic anchor: Hypothalamic BNC2 neurons as key metabolic responders

The functional characterization of hypothalamic BNC2 neurons has provided crucial mechanistic insights into rapid appetite control. Tan et al. (2024) demonstrated that these neurons possess a potent capacity for immediate appetite suppression through monosynaptic GABAergic inhibition of orexigenic AgRP/NPY neurons.^5^ This mechanism illustrates an elegant opponent-process logic: following activation by the satiety hormone leptin, BNC2 neurons release GABA to rapidly silence the brain’s primary feeding drive.^5^

However, the precise biophysical nature of this sensing requires rigorous definition. While Tan et al. proved that deleting the leptin receptor (*Lepr*) specifically in BNC2 neurons leads to hyperphagia and obesity, thereby confirming that these neurons are essential and cell-autonomous targets of leptin, it remains to be determined via electrophysiological recordings under synaptic blockade whether BNC2 neurons are putative first-order sensors (depolarizing immediately upon leptin binding) or integrated responders that summate leptin-driven inputs from other network nodes. Therefore, we refer to them as primary metabolic responders rather than validated autonomous sensors until such evidence is available.

Regardless of the millisecond-scale electrophysiology, the transcriptional primacy of BNC2 is evident. As a transcription factor, BNC2 likely orchestrates this function through a hierarchical mechanism: it may maintain the chromatin accessibility required for the expression of critical synaptic machinery (e.g., GABA synthesis enzymes) or neuropeptide processing genes, thereby setting the excitability tone of these neurons. This positions BNC2 not merely as a participant, but as a foundational regulator that enables the neuron’s metabolic responsiveness.

### Beyond homeostasis: BNC2 in reward and motivation circuits

A comprehensive theory of energy balance must account for both the homeostatic need to eat (hunger) and the hedonic drive to consume palatable foods (craving). A transformative advance in understanding BNC2’s broader role comes from recent findings in the ventral pallidum (VP), a key interface in the limbic reward system.^41^ Unlike the arcuate nucleus, which primarily senses energy deficits, the VP integrates motivation and reward valuation.

Recent study establishes that BNC2-expressing neurons in the VP constitute a genetically distinct population, separate from the classical cholinergic or parvalbumin-positive neurons often associated with this region.^41^^,^^42^ Crucially, the activity of these VP-BNC2 neurons is sensitive to nutritional state, and their specific chemogenetic manipulation significantly alters high-fat diet intake and body weight gain.^41^^,^^42^ This discovery allows us to propose a dual-node coordination model: BNC2 expressing neurons functions as a distributed network hub, regulating homeostatic satiety in the hypothalamus^5^ while simultaneously modulating the incentive salience of food in the ventral pallidum.^41^ This synchronization ensures that metabolic behavior is coherent across both physiological and psychological domains.

### Potential intersections with behavioral state

Beyond feeding behavior, emerging evidence suggests BNC2 may function at the nexus of metabolism and affective state. A formidable challenge in modern medicine is the high comorbidity between metabolic disorders (obesity and diabetes) and psychiatric conditions (depression and anxiety). While speculative at this stage, we propose that BNC2-dependent molecular mechanisms in specific neural circuits could represent a candidate bridge linking these distinct physiological domains.

First, correlative evidence links BNC2 to the developmental programming of emotional circuits. BNC2 is significantly upregulated following BDNF manipulation in the neonatal medial prefrontal cortex, a region critical for emotional regulation, where transient BDNF overexpression leads to anxiety-like behaviors in adolescence.^49^ While a direct causal link remains to be established through loss-of-function studies, this association implies that BNC2 might participate in the molecular machinery bridging metabolic dysfunction and mood disorders. Second, and perhaps more mechanistically relevant to metabolic disease, is the link to immunometabolism. Circular RNA derived from *BNC2* (circ-BNC2) has been shown to regulate neuroinflammation in microglial cells via the miR-497a-5p/*HECTD1* axis.^50^ Since chronic, low-grade neuroinflammation is a recognized driver of both central insulin resistance (type 2 diabetes) and depressive symptoms,^51^ BNC2’s ability to suppress neuroinflammation positions it as a potential neuroprotective factor.

## BNC2 and the canonical metabolic guard: Integration or independence?

As we position BNC2 within the metabolic regulatory landscape, a critical question arises: how does it interface with established giants like STAT3 and FOXO1? These factors are the canonical effectors of leptin and insulin signaling.^2^

BNC2 is unlikely to act merely as a redundant parallel pathway. Structurally, BNC2 is a zinc-finger protein capable of binding specific DNA motifs, whereas STAT3 is a signal transducer activated by phosphorylation. We hypothesize a cooperative hierarchy model.

(1) Temporal segregation: STAT3 phosphorylation provides the immediate transcriptional response to leptin (minutes to hours), while BNC2, acting as a constitutive nuclear factor,^6^^,^^8^ may define the permissive chromatin landscape that dictates which genes are accessible to STAT3. This would position BNC2 as a chromatin competency factor.
(2) Spatial complementarity: While STAT3 is broadly expressed in *LepR*+ neurons, BNC2 expression is more restricted.^52^ This suggests BNC2 may confer regional specificity to the generic leptin signal, ensuring that leptin triggers appetite suppression in the arcuate nucleus but potentially distinct behaviors (e.g., motivation) in the ventral pallidum.

Future studies utilizing ChIP-seq to map overlapping genomic binding sites of BNC2 and STAT3/FOXO1 will be essential to validate this convergent regulation.

## A metabolic integration framework: BNC2 as a putative coordinator

Integrating evidence from hypothalamic^5^ and ventral pallidum circuits,^41^ we propose that BNC2 functions as a putative transcriptional hub for metabolic state integration. This framework posits that BNC2 coordinates the translation of peripheral energy signals into appropriate neural circuit activity, enabling the brain to align behavioral outputs with metabolic needs.

This metabolic integration hypothesis rests on four key pillars.

**Figure 3:**
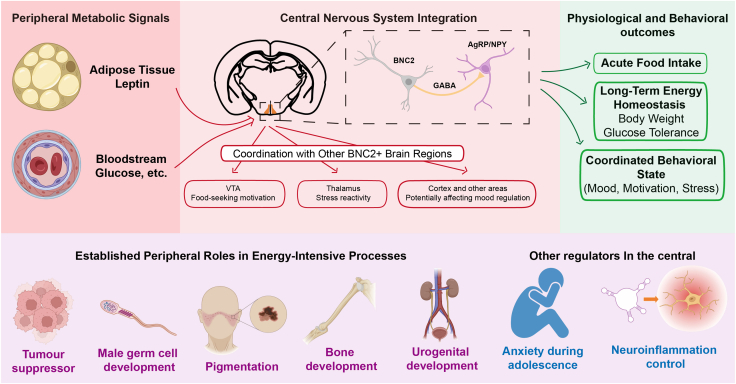
A hierarchical framework positioning BNC2 as a putative metabolic integrator
(2) Deep evolutionary conservation: the remarkable conservation of BNC2 suggests its integrative function is fundamental to vertebrate physiology, making a conserved role from periphery to CNS plausible^6^ (Figure 3).
(3) Strategic metabolic circuit expression: BNC2’s selective expression in hypothalamic energy control centers, reward circuits (ventral pallidum), and stress-metabolic relay nodes positions it ideally for metabolic state integration.
(4) Metabolic responsiveness: hypothalamic BNC2 neurons regulate homeostatic feeding,^5^ while ventral pallidum BNC2 neurons modulate reward-driven intake,^41^ establishing proof-of-concept for metabolic state integration.

(1) A Precedent of adaptive coordination: BNC2’s well-established pleiotropic roles in coordinating development and homeostasis across diverse peripheral tissues set a strong precedent for an integrative function^10^^,^^11^^,^^13^^,^^14^^,^^15^^,^^16^^,^^17^^,^^18^^,^^19^^,^^20^^,^^21^^,^^22^^,^^23^^,^^53^^,^^54^ (summarized in the hierarchical model in Figure 3).

Under this proposed framework, we envision BNC2 could operate as the lynchpin of a hierarchical metabolic coordination network: hypothalamic BNC2 neurons serve as primary energy state responders, integrating leptin, glucose, and other metabolic signals. This metabolic information could then be broadcast to other BNC2-expressing regions to coordinate a suite of state-appropriate behaviors, including modulating food-seeking motivation in the VTA, adjusting stress responsiveness in the thalamus, and potentially influencing mood regulation to maintain energy balance.

This schematic illustrates the proposed role of BNC2 as a transcriptional hub that translates peripheral metabolic signals into coordinated neural and behavioral responses. The framework operates across three functional levels: (1) peripheral input: systemic energy status is conveyed via circulating signals, including adipose-derived leptin and glucose^5^; (2) central integration: in the hypothalamus, BNC2 modulates AgRP/NPY and GABA signaling to gate homeostatic feeding. Concurrently, BNC2 expression in distributed networks (VTA, thalamus, and cortex) is hypothesized to coordinate food-seeking motivation, stress reactivity, and emotional state^5^; (3) systemic output: the integrated response encompasses acute behaviors (appetite suppression), long-term metabolic adaptations (body weight, glucose tolerance), and behavioral alignment (mood, motivation).^5^ lower panel: summary of BNC2’s established roles in resource-intensive peripheral processes (tumor suppression, germ cell development, pigmentation, skeletal, and urogenital maintenance)^10^^,^^11^^,^^13^^,^^14^^,^^15^^,^^16^^,^^17^^,^^18^^,^^19^^,^^20^^,^^21^^,^^22^^,^^23^^,^^53^^,^^54^ alongside emerging central regulatory functions (neuroinflammation control, developmental emotional programming).^49^^,^^50^ This model proposes BNC2 functions as a multi-system coordinator, linking cellular resource allocation with organismal energy balance. Created with BioRender.com.

## Discussion: knowledge gaps and a roadmap for validating BNC2 as a metabolic coordinator

The emerging evidence positions BNC2 as a compelling, albeit putative, candidate for a transcriptional coordinator of energy balance. However, to mature from an intriguing hypothesis into a definitive metabolic framework, several critical limitations in the current literature must be addressed. These knowledge gaps define a clear roadmap for future investigation.

### Isoform specificity and molecular function

As noted, the *Bnc2* gene possesses a remarkable capacity for extensive alternative splicing, theoretically generating a vast number of mRNA isoforms.^7^ Current spatial transcriptomics (MERFISH) and functional studies utilizing constitutive or generic Cre lines do not distinguish which specific splice variants are active in metabolic circuits. Given the high homology within zinc-finger families, there is a risk that probe sets may detect non-functional variants or that different isoforms may have distinct, even opposing, functions in different neuronal populations. Future work must prioritize the use of long-read sequencing technologies (e.g., Iso-seq) coupled with single-cell resolution to identify the functionally relevant transcripts in hypothalamic versus ventral pallidal neurons.

### Specificity of metabolic sensing

While leptin responsiveness of hypothalamic BNC2 neurons has been convincingly demonstrated,^5^ the precise biophysical nature of this sensing requires rigorous definition. It remains to be determined via electrophysiological recordings under synaptic blockade whether these neurons are bona fide first-order sensors or integrated responders that summate leptin-driven inputs from other network nodes. A true metabolic nexus would also imply integration of multiple signals (e.g., insulin, ghrelin, and GLP-1), a possibility that has yet to be explored for BNC2 neurons in any brain region. Future studies should combine snap-shot chemogenetics with *in vivo* fiber photometry or electrophysiology to dissect the direct versus indirect nature of hormonal sensing by these cells.

### Developmental compensation and circuit-level connectivity

Much of the existing phenotypic data, particularly from peripheral tissues, relies on developmental or constitutive manipulations. To firmly establish a role in the adult regulation of energy balance, it is essential to validate these findings with adult-inducible, cell-type-specific deletion systems. This approach would rule out confounding effects from developmental compensation. Furthermore, while we have identified key nodes of expression, the connectivity of these nodes remains largely unknown. A high-priority goal is to use modern viral tracing tools, such as monosynaptic rabies tracing,^55^ to delineate the full input-output organization of BNC2 neuron networks in the hypothalamus, ventral pallidum, and thalamus.

### Platform-dependent validation and human relevance

The current reliance on MERFISH data for expression mapping is powerful, but it requires orthogonal validation. Our cross-reference with bulk RNA-seq data provides initial support, yet quantitative validation using independent methods (e.g., RNAscope, or interrogation of emerging single-cell RNA sequencing atlases) is necessary to statistically confirm the enrichment patterns observed. Finally, the ultimate test of this framework’s relevance lies in humans. Future work should leverage large-scale GWAS datasets to investigate whether *BNC2* genetic variants are associated not only with obesity, but also with hedonic eating traits, food addiction, and the well-documented comorbidity between obesity and depression.

In conclusion, BNC2 has emerged as a promising player in brain circuits controlling fundamental metabolic behaviors. The discovery of its roles in both the hypothalamus and ventral pallidum provides a strong foundation for a new distributed coordinator hypothesis. By systematically addressing the critical gaps outlined previously, deciphering isoform-specific functions, mapping circuit connectivity, validating direct metabolic sensing, and establishing human translational relevance, the field can transition from observation to mechanistic validation. Deciphering the BNC2 regulatory network ultimately offers a fascinating new perspective on how the brain integrates energy state with behavior, potentially unlocking therapeutic paradigms for obesity and its related metabolic and affective disorders.

### Materials availability

This study is a perspective analysis of publicly available datasets and did not generate new unique reagents or biological materials. All data associated with this study are available in the article or the online supplemental information.

### Data and code availability

Data requests should be directed to and will be fulfilled by the lead contact. The study in this article does not report original code. All visualizations were performed using the built-in tools of the respective databases or standard graphing software (GraphPad Prism). Additional information will be made available by the lead contact upon request.

## Acknowledgments

We thank the Allen Institute for Brain Science for providing open access to the Mouse Whole-Brain Transcriptomic Cell Type Atlas (MERFISH, Allen Brain Cell Atlas; knowledge.brain-map.org), and the Human Protein Atlas consortium for publicly available bulk RNA-seq data (www.proteinatlas.org). We also acknowledge Dr. Joann B. Sweasy and colleagues for helpful discussions and constructive suggestions during the preparation of this manuscript. Schematic illustrations (Figure 3) were created with BioRender.com. This work was supported by Beijing Life 10.13039/501100008967Science Academy (2024600CB0130), Qingdao Municipal Bureau of Science and Technology (25-1-5-xdny-19-nsh), the Agricultural Science and Technology Innovation Program of Chinese Academy of Agricultural Sciences (ASTIP-TRIC05), and the Medical Specialty Ascent Program of Southeast University (4024002407, awarded to M.M.).

## Author contributions

Writing the original draft, visualization, investigation, and methodology, Z.Z.; writing the original draft, visualization, investigation, and methodology, S.H.; review and editing, K.H.; review and editing, P.L.; review and editing, Y.D.; review and editing, W.S.; review and editing, S.S.; review and editing, supervision, and funding acquisition, H.Z.; conceptualization, supervision, writing the original draft, review and editing, and funding acquisition, M.M.; contributed equally to this work, Z.Z., S.H., and M.M. All authors read and approved the final manuscript.

## Declaration of interests

The authors declare no conflicts of interest.
